# Untangling Alzheimer’s disease with spatial multi-omics: a brief review

**DOI:** 10.3389/fnagi.2023.1150512

**Published:** 2023-07-17

**Authors:** Cody R. Marshall, Melissa A. Farrow, Katerina V. Djambazova, Jeffrey M. Spraggins

**Affiliations:** ^1^Chemical and Physical Biology Program, Vanderbilt University School of Medicine, Nashville, TN, United States; ^2^Mass Spectrometry Research Center, Vanderbilt University School of Medicine, Nashville, TN, United States; ^3^Department of Biochemistry, Vanderbilt University School of Medicine, Nashville, TN, United States; ^4^Department of Cell and Developmental Biology, Vanderbilt University School of Medicine, Nashville, TN, United States; ^5^Department of Chemistry, Vanderbilt University, Nashville, TN, United States

**Keywords:** Alzheimer’s disease, spatial multi-omics, mass spectrometry, neuroscience, imaging mass spectrometry (IMS), spatial transcriptomics (ST), microscopy, immunoflorescence

## Abstract

Alzheimer’s disease (AD) is the most common form of neurological dementia, specified by extracellular β-amyloid plaque deposition, neurofibrillary tangles, and cognitive impairment. AD-associated pathologies like cerebral amyloid angiopathy (CAA) are also affiliated with cognitive impairment and have overlapping molecular drivers, including amyloid buildup. Discerning the complexity of these neurological disorders remains a significant challenge, and the spatiomolecular relationships between pathogenic features of AD and AD-associated pathologies remain poorly understood. This review highlights recent developments in spatial omics, including profiling and molecular imaging methods, and how they are applied to AD. These emerging technologies aim to characterize the relationship between how specific cell types and tissue features are organized in combination with mapping molecular distributions to provide a systems biology view of the tissue microenvironment around these neuropathologies. As spatial omics methods achieve greater resolution and improved molecular coverage, they are enabling deeper characterization of the molecular drivers of AD, leading to new possibilities for the prediction, diagnosis, and mitigation of this debilitating disease.

## Introduction

Alzheimer’s disease (AD) is the most common form of neurological dementia, with an estimated 6.07 million adults in the United States diagnosed with the disease by 2020 and a predicted 13.85 million adults by 2060 (Rajan et al., 2021). A progressive disability, initial symptoms of AD include mild memory loss, followed by gradually increasing symptoms of disorientation, mood and behavior change, and deepening confusion while the disease spreads throughout regions of the brain (Rajan et al., 2021). The characteristic neuropathologies associated with AD include neuritic plaques consisting of extracellular β-amyloid deposits and neurofibrillary tangles (NFTs) composed of aggregated hyperphosphorylated tau protein (p-tau) (Long and Holtzman, 2019). Unfortunately, most drug candidates in the last few decades targeting β-amyloid and p-tau failed to show clinical efficacy. One exception is lecanemab, a monoclonal antibody that targets β-amyloid and modestly slows AD progression, which was recently approved by the FDA. However, there are still no preventative treatments for AD (van Dyck et al., 2022). The failure of many of these putative therapeutic candidates coincides with the lack of understanding of the molecular underpinnings of AD onset and progression. Therefore, more research into the complex and heterogeneous nature of AD is necessary to better treat this disease.

There are several emerging but less-understood AD-associated pathologies, including cerebral amyloid angiopathy (CAA), dystrophic neurites, neuropil threads, granulovacuolar degenerating bodies, and gliosis (Long and Holtzman, 2019). CAA occurs when toxic β-amyloid deposits form within cerebral arterioles and capillaries in the central nervous system leading to cognitive impairment and intracerebral hemorrhage (Biffi and Greenberg, 2011; van Etten et al., 2014; Boyle et al., 2015). Dystrophic neurites contain an accumulation of dysfunctional lysosomes within distending axons that surround β-amyloid plaques, while granulovacuolar degenerating bodies are pathologic neuronal organelles with lysosome-related proteins thought to be clogged autophagosomes (Schrag et al., 2020). In addition, astrocytes and microglia surrounding neural tissue demonstrate forms of gliosis in AD. Despite the varied molecular and cellular drivers of these localized sites of dysfunction, each is highly correlated with AD. For example, CAA is reported to occur in 82 to 98% of AD patients (Attems and Jellinger, 2014). Additional studies have demonstrated that CAA correlates to neurodegenerative disease and cognitive impairments, more generally (Debette and Markus, 2010; Schrag et al., 2010; Carare et al., 2014; Lee et al., 2018). Researchers also suggest that defective neuronal endolysosomes accumulating within dystrophic neurites surrounding β-amyloid plaques might have a molecular interaction with the autophagic organelles in granulovacuolar degenerating bodies (Schrag et al., 2020). While these contributory pathologies are observed frequently among patients with AD, the heterogeneous nature and interactions between them remain unclear. Traditional multi-omic strategies, which include integrated genomics, epigenomics, transcriptomics, proteomics, and metabolomics/lipidomics studies, have identified AD-associated molecular drivers and biomarkers of the neuropathologies mentioned above (Badhwar et al., 2020; Johnson et al., 2020; Clark et al., 2021, 2022; François et al., 2022; Gao et al., 2022; Kodam et al., 2023). Still, future analyses must discern the spatial relationship and interconnectedness between AD biomarkers, cell types, and contributing pathologies in order to develop more precise therapeutics. Recent technological advances in spatial omics can illuminate these meaningful relationships across time and space. This review will focus on novel spatial multi-omics technologies, comparing spatial molecular profiling strategies with molecular imaging (See Table 1). Close attention will be paid to imaging mass spectrometry (IMS) as a modality primed to help untangle the spatial complexity of Alzheimer’s disease.

**TABLE 1:** Spatial omics technologies.

	Technique	Resolution	Molecular class	Molecular coverage	References
***Spatial Profiling***	LCM-MS	10–50 μm	Proteins, lipids, metabolites	1,000s	Datta et al., 2015; Knittelfelder et al., 2018
	NanoPOTS	50 μm	Proteins, metabolites	100s	Xu et al., 2019
	microLESA	80 μm	Proteins, metabolites	100s	Ryan et al., 2019; Guiberson et al., 2021
	LCM-seq	10–50 μm	RNA	Transcriptome wide	Nichterwitz et al., 2018
	GeoMx	0.5–2 μm	Proteins, RNA	100s, Transcriptome wide	Merritt et al., 2020
***Molecular Imaging***	CycIF	0.5-2 μm	Proteins	10s	Muñoz-Castro et al., 2022
	MP-IHC	0.5–2 μm	Proteins	100s	Murray et al., 2022
	Immuno-SABER	0.5–2 μm	Proteins	10s	Saka et al., 2019
	CODEX	0.5–2 μm	Proteins	10s	Goltsev et al., 2018; Black et al., 2021; Hickey et al., 2022; Neumann et al., 2022
	MIBI-TOF	0.5–1 μm	Proteins	10s	Vijayaragavan et al., 2022
	MALDI IMS	5–40 μm	Proteins, lipids, metabolites	100s	Spengler and Hubert, 2002; Norris and Caprioli, 2013; Michno et al., 2018; Spraggins et al., 2019; Michno et al., 2022
	DESI IMS	30–75 μm	Proteins, lipids, metabolites	100s	Ifa et al., 2010; Eberlin et al., 2011; Chew et al., 2020; Qi et al., 2021
	Nano-DESI IMS	12–75 μm	Proteins, lipids, metabolites	100s	Roach et al., 2010
	SIMS	0.1–10 μm	Proteins, lipids, metabolites	100s	Wu and Odom, 1996; Heeren et al., 2006; McDonnell et al., 2006; Lazar et al., 2013
	Slide-seqV2	10 μm	RNA	Transcriptome wide	Rodriques et al., 2019; Stickels et al., 2021
	MERFISH	0.5–2 μm	RNA	Transcriptome wide	Chen et al., 2015
	Visium	10–50 μm	RNA	Transcriptome wide	Lebrigand et al., 2023

## Tools for spatial omics I: spatial profiling

Understanding the complex and concerted molecular drivers of diseases such as AD requires insight into how cellular neighborhoods and molecular distributions are altered near sites of dysfunction. Spatial profiling experiments achieve this by performing omics measurements on specific tissue structures, cell types, and/or single cells through discrete surface sampling approaches (Moffitt et al., 2022). These spatially derived samples are then analyzed using advanced omics technologies, including next-generation sequencing or liquid chromatography-mass spectrometry (LC-MS), Moffitt et al. (2022) producing generate deep molecular profiles (i.e., hundreds to thousands of molecular species) that span a wide range of molecular classes. Profiling experiments often sacrifice spatial information by collecting data from larger tissue areas (>100 μm^2^) or from dispersed cells to allow for enough material to maximize molecular coverage, sensitivity, and dynamic range. This contrasts with imaging approaches that provide more complete spatial distributions at higher resolution, often with diminished molecular coverage and sensitivity. For Alzheimer’s disease, this approach can be used target neuropathological foci such as neuritic plaques or NFTs to understand better how these features change cellular interactions and biomolecular mechanisms *in situ*.

Spatial profiling methods can be coupled to proteomics workflows to determine the abundance of proteins produced or modified at specific locations in tissue. Often spatial proteomics experiments are performed in tandem with complementary transcriptomics experiments allowing for relationships between gene expression and protein abundance to be better understood (Gry et al., 2009). Spatial proteomics utilizing mass spectrometry (MS) is advantageous over other approaches, such as antibody-based techniques, because it enables untargeted, rapid, highly specific analysis of thousands of proteins and proteoforms (Bai et al., 2021). Additionally, it does not require any *a priori* information. It is optimal for detecting post-translational modifications (PTMs) that often cannot be observed with antibody protein labeling techniques, which bind to a specific epitope (Bai et al., 2021). These highly specific MS-based methods are important when assessing molecular heterogeneity associated with neuropathologies as PTMs have been found to play an important role in Alzheimer’s disease (Deture and Dickson, 2019). For example, hyperphosphorylated tau is a hallmark feature of neuropil threads and neurofibrillary tangles, which correlate to disease severity (Giannakopoulos et al., 2003). One strategy for producing spatially resolved LC-MS results is to utilize laser capture microdissection (LCM). This is a standard method that uses a cutting laser to isolate discrete tissue regions (>10 μm in diameter) for MS analysis (Datta et al., 2015). Isolated tissue regions are then prepared and analyzed using of proteomics, lipidomics, and metabolomics (Drummond et al., 2018; Knittelfelder et al., 2018). In one study, researchers quantified ∼900 proteins in plaques and ∼500 proteins in NFTs in human Alzheimer’s tissue using LCM followed by LC-MS (Hughes et al., 2014). Recent advances have been made to maximize tissue collection efficiency for LCM experiments, improving the overall sensitivity and molecular coverage of the workflow. Many of these new methods utilize single tubes or droplets for sample collection and processing, which minimizes sample loss and reduces the total amount of tissue needed for the subsequent analyses (Hughes et al., 2014; Kulak et al., 2014; Moggridge et al., 2018). Techniques like NanoPOTS (Nanodroplet Processing in One pot for Trace Samples) employ a robotic/microfluidic platform that collects tissue and performs processing steps in ∼200 nL droplets in order to improve overall sensitivity and map protein expression at higher spatial resolutions resolution (Xu et al., 2019). Another profiling approach used to identify proteins in a particular tissue region while maintaining spatial integrity is micro-liquid extraction surface analysis (microLESA) (Ryan et al., 2019; Guiberson et al., 2021). This technique utilizes a piezo-electric spotter to deposit nanoliter droplets of trypsin onto defined tissue regions, allowing for bottom-up proteomics to be performed from foci as small as 80 μm (Ryan et al., 2019; Guiberson et al., 2021). MicroLESA can be performed more rapidly than LCM but with reduced spatial resolution. The trajectory of spatial molecular profiling toward smaller detection areas puts these technologies in a unique position to discover altered pathways and signaling networks associated with neuropathogenic structures like neuritic plaques and NFTs, which require cell-type or even single-cell differentiation.

**FIGURE 1:**
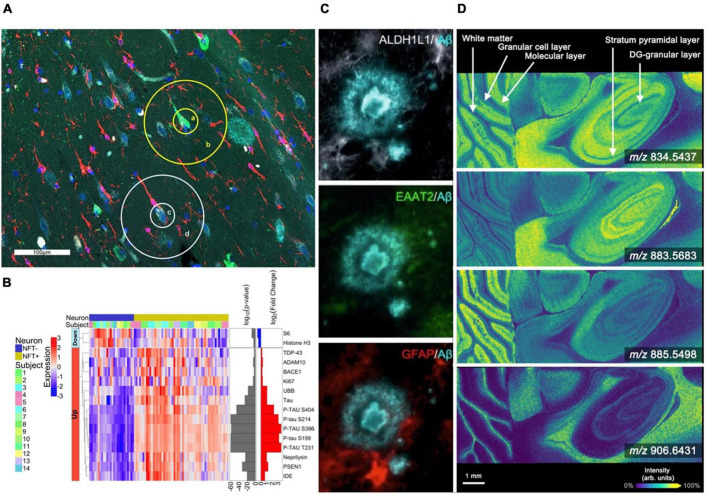
Spatial profiling and imaging technologies used in brain studies. **(A,B)** The GeoMx platform allows for highly multiplexed analysis of gene and protein expression [modified from Walker et al. (2022)]. **(A)** The Hippocampal CA1 subregion labeled with morphology markers (AT8, green; β-amyloid, aqua; IBA-1, red; nuclear marker SYTO13, blue) to highlight targeted NFTs and surrounding areas. **(B)** Heatmap of differentially expressed proteins when comparing NFTs to normal neurons (*p* < 0.01). **(C)** Representative highly multiplexed CycIF images of astroscytes from a AD donor highlighting ALDH1L1, EAAT2, GFAP, and Aβ [modified from Muñoz-Castro et al. (2022)]. **(D)** MALDI imaging mass spectrometry lipid data from murine brain collected at 10 μm spatial resolution. Selected images include *m/z* 834.5437 ([PS(40:6)-H]^–^, –0.12 ppm error), *m/z* 883.5683 ([PI(38:5)-H]^–^, –0.45 ppm error), *m/z* 885.5498 ([PI(38:4)-H]^–^, –0.23 ppm error), and *m/z* 906.6431 ([SHexCer(t42:1)-H]^–^, –1.9 ppm error) [modified from Spraggins et al. (2019)].

There are also emerging profiling-based spatial transcriptomics technologies that provide insights into localized gene expression in targeted cells and tissue features (Lovatt et al., 2014; Nichterwitz et al., 2018; Chen et al., 2020; Merritt et al., 2020; Qian et al., 2020; Rao et al., 2021; Walker et al., 2022). Traditional single-cell and single-nuclei RNA sequencing approaches (scRNA-seq/snRNA-seq) enable cell phenotyping by generating deep molecular information for individual cells but do not provide spatial context (Rao et al., 2021). LCM coupled with sequencing workflows (LCM-seq) offers the ability to target tissue regions while allowing for the flexibility to perform most sequencing approaches. For example, LCM-seq has been used to perform polyA-based RNA sequencing on individual neurons, preserving information regarding the location of each cell relative to the tissue environment (Nichterwitz et al., 2018). Similar to proteomics, this approach has challenges associated with tissue loss and collection efficiency during the LCM process. A different tissue profiling approach has been commercialized by NanoString using a platform called the GeoMx digital spatial profiler (Merritt et al., 2020). This technology uses RNA probes bound to oligo barcodes using a UV-cleavable linker. Probes are hybridized to endogenous RNA in tissue sections, the barcodes are released from targeted tissue regions using focused UV light, and those barcodes are aspirated and sequenced using next-generation sequencing. When coupled with immunohistochemistry, this technology allows for highly precise and accurate targeting of specific cell types and tissue features while assessing expression for >18,000 genes in human and murine tissues. The GeoMx digital spatial profiler is already being employed in the study of AD. For example, Walker et al. (2022) used a cocktail of 86 antibodies conjugated to unique UV-photocleavable oligonucleotide tags, allowing researchers to determine differential gene expression in hippocampal regions between dementia patients with AD neuropathological changes and cognitively normal patients (termed resilient) with AD neuropathological changes (Figures 1A, B). Although powerful, this approach is limited to studies of species of which probe sets are available. Currently, nanoString only offers probe sets for the human and mouse genome. Overall, transcriptomic spatial profiling techniques have advanced significantly over a short period of time. They will undoubtedly become more prominent in understanding transcript-level alterations at sites of AD-associated pathologies. With the ability to analyze a wide range of molecular classes and link observations to specific tissue features and cell types, spatial molecular profiling technologies are well-positioned to help decipher the complexity associated with AD and related diseases.

## Tools for spatial omics II: molecular imaging

Molecular imaging combines the spatial characteristics of traditional histology with the molecular specificity of modern omics technologies. Although imaging approaches provide reduced sensitivity and coverage compared to bulk or profiling measurements, they more completely capture the spatial context of biomolecular processes *in situ*. This is critically important for complex diseases like AD as localized disorder of molecular and cellular neighborhoods often drive disease pathology (Mrdjen et al., 2019).

Revealing spatial distributions of proteins in biological tissue is traditionally driven by antibody-based technologies, including immunohistochemistry (IHC) and immunofluorescence (IF), providing the ability to map targeted proteins at high spatial resolution (Weissleder, 2002; Goltsev et al., 2018; Saka et al., 2019; Black et al., 2021; Hickey et al., 2022; Murray et al., 2022; Muñoz-Castro et al., 2022; Neumann et al., 2022; Wang et al., 2022). IF allows the detection of multiple protein targets, but plexity is typically limited to ∼4 to 7 markers due to spectral overlap of the emission spectra for individual fluorochromes (Tsurui et al., 2000). However, new approaches have been developed that enable highly multiplexed antibody-based imaging. Cyclic IF (CycIF) uses cycles of antibody staining and stripping to produce high plexity molecular images, typically with 3–5 markers in each cycle. An 8-cylce cyclic IF workflow allowed researchers to discover a new third state of glial cells found near plaques and tangles (Muñoz-Castro et al., 2022; Figure 1C). This state was hypothesized to be an “intermediate” cell population between homeostatic and reactive microglia. Murray et al. (2022) have developed an optimized CycIF workflow for brain tissue using commercially available primary and secondary antibodies termed multiplexed fluorescence-based immunohistochemistry (MP-IHC). They used this approach to screen 100 markers in the olfactory bulb from post-mortem tissues of Alzheimer’s disease and Parkinson’s disease patients. Leveraging pixel-wise unsupervised machine learning, the resulting multiplexed imaging data were used to segment anatomical features and group samples based on disease type (Murray et al., 2022). The ability to distinguish specific cell types and states is essential for understanding how cellular neighborhoods are altered at sites of AD neuropathology. However, most CycIF strategies are performed manually and are prone to tissue loss during the cycling steps.

Researchers are also working to improve robustness and throughput for multiplexed protein imaging. Although yet to be applied to the study of AD, one approach that is gaining traction is co-detection by indexing (CODEX) IF microsocopy (Goltsev et al., 2018; Saka et al., 2019; Black et al., 2021; Hickey et al., 2022; Neumann et al., 2022). CODEX is performed using DNA-conjugated antibodies and then cycling the fluorescently labeled complementary oligo barcodes allowing for >50 markers to be imaged from a single tissue section. One drawback of CODEX is the preparation and validation required to conjugate primary antibodies. However, the process is less prone to tissue loss and more amenable to cycling, increasing the number of markers that can be imaged reproducibly. There are also MS-based approaches that use antibodies labeled with elemental mass reporters, such as cytometry time-of-flight (CyTOF) (Wang et al., 2022) imaging and multiplexed ion beam imaging by time-of-flight (MIBI-TOF) (Vijayaragavan et al., 2022). With MIBI, a high-energy ion beam is used to sample the tissue surface, sputtering elemental reporters, and detecting the reporters using a TOF mass spectrometer at each pixel location. Using MIBI, authors validated and imaged 36 brain-abundant targets while comparing healthy and AD hippocampus tissue regions (Vijayaragavan et al., 2022). The results of this study identified persistent neurons expressing MFN2 that were adjacent to NFTs, suggesting that this mitochondrial membrane protein carries a survival advantage for neurons evading tau-induced pathology (Vijayaragavan et al., 2022). MS-based technologies allow for higher plexity within a single scan without the challenges of overcoming overlapping fluorescence emission bands. However, these approaches are typically lower throughput, limited to only imaging small tissue areas, and completely ablate the tissue eliminating the ability for subsequent experiments. On the other hand, fluorescence microscopy-based approaches require complex cycling methods to achieve high plexity but allow for whole-slide imaging.

Imaging mass spectrometry (IMS) is another multiplexed molecular imaging technology that enables untargeted mapping of proteins, glycans, lipids, and metabolites (Caprioli et al., 1997; Spengler and Hubert, 2002; Gode and Volmer, 2013; Norris and Caprioli, 2013; Wu et al., 2013; Nilsson et al., 2015; Spraggins et al., 2019; Djambazova et al., 2020, 2023). This label-free approach can simultaneously detect 100s to 1,000s of endogenous biomolecules in a pixel-wise manner, generating molecular tissue maps (Caprioli et al., 1997). Although numerous IMS technologies exist, matrix-assisted laser desorption/ionization (MALDI) and desorption electrospray ionization (DESI) have most extensively been applied to AD. MALDI IMS utilizes an applied chemical matrix and laser ablation to desorb and ionize analyte molecules from tissue (Norris and Caprioli, 2013; Figure 1D). Significant advances in laser and stage technologies have allowed for higher spatial resolution (≤10 μm pixel size) (Spraggins et al., 2019), in some cases ablating tissue areas of less than 1 μm (Spengler and Hubert, 2002). This makes MALDI IMS a powerful tool for studying changes associated with Alzheimer’s disease at cellular resolution. Recent studies targeting the tissue microenvironment of amyloid plaques have observed various molecular signatures, distinct lipid accumulations, and even monitored Aβ-peptide content within and around β-amyloid plaques (Casadonte et al., 2015; Kaya et al., 2018; Michno et al., 2018, 2021, 2022; Kelley et al., 2020; Koutarapu et al., 2022). The Hanreider group used MALDI combined with plaque staining to identify molecular heterogeneous plaques in a mouse model of AD and identified differential lipid signatures across multiple stages of plaque development (Michno et al., 2022). For example, the data revealed inositol phospholipids, lyso-phosphatidylinositols, and ganglioside (i.e., GM2 and GM3) lipids localized to immature AD plaques. Whereas phosphatidylethanolamines and phosphatidic acids were found in higher abundance in the core regions of mature plaques. Their research has revealed the vast molecular heterogeneity during plaque development. DESI IMS is another common ionization source for performing IMS experiments. DESI is performed by spraying charged droplets onto the tissue surface, which dissolve endogenous molecules and are deflected into the mass spectrometer inlet (Ifa et al., 2010; Eberlin et al., 2011). This approach allows for rapid analysis due to not requiring specific sample preparations; however, spatial resolution is typically limited to 30–50 μm pixel sizes (Qi et al., 2021). DESI IMS is especially effective for imaging lipids (Roach et al., 2010; Chew et al., 2020; Zhang et al., 2021), which are implicated in the progression of Alzheimer’s disease. One study incorporating DESI IMS detected significant changes in glycerophospholipid metabolism in correlation with the early stages of AD (Zhang et al., 2021). Finally, secondary ion mass spectrometry (SIMS) (Wu and Odom, 1996) is being utilized for high resolution molecular imaging studies of AD (Lazar et al., 2013). SIMS utilizes a focused ion beam allowing for pixel sizes of 100 nm–1 μm (Wu and Odom, 1996). However, depending on the ion beam used, fragmentation of endogenous molecules can occur during desorption and ionization, typically limiting SIMS analysis to low molecular weight species (Heeren et al., 2006). One example application of SIMS to AD found heterogeneous distributions of cholesterol in the cerebral cortex (Lazar et al., 2013). They found a significant increase in cholesterol signal in the external granular layer/external pyramidal layer and the internal granular layer (Lazar et al., 2013). Advances to SIMS, such as surface modification, matrix-enhanced SIMS and metal-assisted SIMS, and polyatomic ions, are helping to expand the applicability of SIMS to a broader range of molecular classes (Wu and Odom, 1996; Heeren et al., 2006; McDonnell et al., 2006).

Similar to profiling technologies, novel imaging approaches have been developed for decoding localized gene expression in relation to cell types and tissue features (Chen et al., 2015; Rodriques et al., 2019; Stickels et al., 2021; Lebrigand et al., 2023). Techniques such as Slide-seq and Slide-seqV2 provide genome-wide expression information at high spatial resolution by capturing RNA onto a surface covered with DNA-barcoded microbeads (Rodriques et al., 2019; Stickels et al., 2021). This approach has been used to map gene expression in the cerebellum and hippocampus from murine brain tissues (Rodriques et al., 2019; Stickels et al., 2021). Another useful method for imaging RNAs is multiplexed error-robust fluorescence *in situ* hybridization (MERFISH), which detected ∼1,000 RNA species within a single cell (Chen et al., 2015). Sequential imaging details genes that are co-expressed and co-regulated in various tissue features. This technique was applied to the primary motor cortex of a mouse, encompassing the transcriptomic information of 300,000 cells and their spatial orientations. Furthermore, *in situ* sequencing technologies are being advanced by commercial vendors like 10x genomics, whose Visium platform performs Spatial Isoform Transcriptomics (SiT), which was able to collect full-length transcript sequences from mouse brain tissue while maintaining spatial context (Lebrigand et al., 2023). Many research groups are working to improve sensitivity, molecular coverage, and spatial resolution for transcriptomic imaging. As spatial transcriptomics technologies advance, these innovative imaging strategies will undoubtedly be applied to AD and associated pathologies to better understand how cellular neighborhoods and biomolecular pathways are altered in normal aging and diseased tissues.

## Conclusion and perspective

The rapid development of spatial molecular profiling and imaging technologies gives scientists a new toolbox for addressing complex diseases at a systems level. Elucidating the interconnectedness between the cellular and molecular organization of neuropathologies associated with Alzheimer’s disease have the potential to bring about breakthroughs in the prediction, detection, and treatment of AD. These breakthroughs are being driven by improvements in the microscopy, mass spectrometry, and transcriptomics assays described here, but advancements are being accelerated through the development of integrated technologies that synergistically combine these assays into multimodal workflows. Multimodal spatial omics experiments provide the ability to understand relationships between a broad range of molecular classes (e.g., RNAs, proteins, lipids, and metabolites) across a wide range of spatial scales (e.g., whole organs to single cells). This will enable a deeper understanding of how cellular neighborhoods and molecular distributions are reorganized around specific neuropathologies found in different tissue microenvironments. In addition to advanced analytical capabilities, this requires novel computational approaches for processing, integrating, and analyzing data from multiple imaging modalities. The future of Alzheimer’s disease research will certainly rely heavily on machine learning to integrate and mine information-rich molecular imaging technologies in both 2- and 3-dimensional space.

Computational strategies to integrate multi-omic information are already providing novel insights into the molecular underpinnings of AD. One study combined transcriptomics, proteomics, and epigenomics approaches to reveal histone modifications specific to AD (Nativio et al., 2020). Using STRING (Search Tool for Retrieval of Interacting Genes/Proteins) analysis, which draws from a protein-protein interaction database and is visualized using the Cytoscape software, researchers showed how epigenetic dysregulation could be a target for early stage AD prevention (Nativio et al., 2020). Taga et al. (2020) used regression analysis to integrate immunohistochemistry data from post-mortem brain tissue with mRNA expression levels. They discovered that BIN1 protein isoforms are differentially expressed in neuronal cell types. In particular, decreased expression of BIN1 isoforms containing exon 7 is associated with a greater accumulation of tangles and subsequent cognitive decline. Researchers have also integrated spatial omics to improve spatial resolution, which is especially important for analyzing smaller features of AD, like plaques and neurofibrillary tangles. One study utilizing a supervised machine learning method called deep data fusion integrated spatial transcriptomics and histology, which allowed for the characterization of the transcriptome of a mouse olfactory bulb on a micrometer scale (Bergenstråhle et al., 2022). Analogous approaches have been applied to IMS data to spatially “sharpen” ion images through data-driven image fusion with microscopy using highly multivariate linear regression (Van de Plas et al., 2015). This method can predict ion distributions with up to 10× higher spatial resolution and in tissue areas not measured by IMS. In the context of AD, where pathological features like plaques and NFTs are on the order of 1–20 um, achieving cellular resolution with the aid of computational tools like image fusion will be extremely valuable. Other supervised machine learning approaches are being used for classification tasks to allow for data-driven recognition of diseased tissues (Lazova et al., 2012; Meding et al., 2012; Hanselmann et al., 2013; Casadonte et al., 2014; Veselkov et al., 2014; Verbeeck et al., 2020). Tideman et al. (2021) recently have shown that supervised and interpretable machine learning could be used for biomarker discovery when applied to untargeted, highly multiplexed MALDI IMS datasets, including for central nervous system tissue. On the other hand, unsupervised machine learning with factorization, clustering, and manifold learning methods are being used for exploratory analysis and interpretation of high dimensionality spatial omics datasets (Verbeeck et al., 2020).

Spatial profiling and imaging is a rapidly expanding field that will continue to see growth. On one end, assays will see further developments as techniques such as imaging mass spectrometry and spatial transcriptomics will achieve greater resolution and become capable of detecting a wider range of molecules. On the other end, data integration and mining with machine learning are now regularly deployed and becoming increasingly necessary for working with large-scale multimodal molecular imaging data. The ability to capture relationships between data modalities in mathematical models and open them for biological interpretation will offer new opportunities for spatially specific biomarker discovery. Using the spatial dimension to connect multi-omic studies will these integrated technologies to be harnessed to reveal how cellular organization and molecular distributions are altered during AD. This will be critical for untangling the mechanisms underlying the diverse array of AD-associated neuropathologies and informing future therapeutic strategies.

## Author contributions

CM, MF, KD, and JS conceptualized and wrote the manuscript. All authors contributed to the article and approved the submitted version.
